# Rapid Nondestructive Testing Technology-Based Biosensors for Food Analysis

**DOI:** 10.3390/bios13050521

**Published:** 2023-05-06

**Authors:** Yong-Huan Yun, Jiangbo Li

**Affiliations:** 1 Key Laboratory of Tropical Fruits and Vegetables Quality and Safety for State Market Regulation, School of Food Science and Engineering, Hainan University, Haikou 570228, China; 2 Intelligent Equipment Research Center, Beijing Academy of Agriculture and Forestry Sciences, Beijing 100097, China

Food analysis plays a vital role in ensuring the safety and quality of food products. Traditionally, food analysis is performed through destructive testing, which involves the physical or chemical alteration of the sample. In recent years, there has been a significant increase in the demand for food safety, quality, and authenticity. To meet this demand, rapid nondestructive testing (RNT) technology has become an important tool in the food industry. It refers to techniques that can analyze the properties of a sample with the advantages of real-time and in-situ analysis, without causing any damage or alteration to the sample.

Biosensors are analytical devices that employ chemical and biological components to detect and quantify the existence or concentration of specific substances in a given sample. They have diverse applications throughout the stages of food production, processing, and storage, enabling quality control, contamination detection, and hazard identification. Over the past few years, significant progress has been made in the development of biosensors in RNT technology for food analysis. Researchers have explored various detection methods, such as electrochemical, optical, and immunological, to develop biosensors that can detect specific food contaminants, including pathogens, pesticides, and mycotoxins, with high precision and sensitivity. Moreover, biosensors have also been developed to detect food quality parameters, such as nutritional components, freshness, ripeness, spoilage, and authenticity. These biosensors can help food producers and processors monitor the quality of their products, reduce waste, and increase the shelf life of the food.

This Special Issue, entitled “Rapid Nondestructive Testing Technology-Based Biosensors for Food Analysis,” includes ten research articles covering electrochemical sensors, colorimetric biosensors, near-infrared (NIR) spectroscopy, fluorescence spectroscopy (FS), and hyperspectral imaging (HSI). By integrating sensor data with chemometric algorithms, researchers have been able to achieve precise detection of various food quality, safety, and authenticity parameters, including freshness, nutritional components, authenticity, pesticide residue, heavy metals, fungal infection, and browning due to physiological factors.

Electrochemical sensors are devices that use the interaction between a chemical species and an electrode to produce an electrical signal, and are powerful tools for detecting and quantifying chemical species in food analysis. Electronic nose and tongue are two types of electrochemical sensors that are commonly used in food analysis to detect and identify the volatile compounds and taste components of food samples. Ren et al. [1] used miniature NIR spectroscopy and electronic tongue sensors, combined with data fusion strategies and chemometric tools, for the taste quality assessment and prediction of multiple grades of black tea, with the ant colony optimization–support vector machine (ACO–SVM) model providing the highest classification accuracy. Zhao et al. [2] proposed a novel approach to detect fungal infections in apples using a portable electronic nose and various machine learning models, including the sparrow search algorithm–backward propagation neural network (SSA–BPNN), with a high recognition accuracy of 98.40%. The study is valuable for the application of electronic noses in the nondestructive and rapid detection of fungal infections in apples. In addition, Peng et al. [3] presented a molecularly imprinted electrochemical sensor for the rapid detection of cyromazine residues in fruits and vegetables. The sensor demonstrated good linearity and reproducibility, with a lower limit of detection of 0.5 µmol/L. This study provides a basis for the development of methods for detecting pesticide residues in edible agricultural products. Lian et al. [4] discussed the optimization of data transmission paths for pest monitoring based on the genetic algorithm (GA), particle swarm optimization (PSO), and simulated annealing (SA). The results show that the optimized path based on PSO can use the least amount of time for data transmission. The study provides a reference for improving the transmission efficiency of agricultural pest monitoring data and developing real-time and effective pest control strategies.

Colorimetric biosensors are a type of biosensor that produces a measurable color change in response to a specific analyte. When combined with a smartphone, the camera can be used to detect and quantify the color change, making it a portable and convenient method for on-site detection of various substances in food analysis. Gu et al. [5] presented a real-time detection method for Hg^2+^ in drinking water using a smartphone as a low-cost micro-spectrometer. The detection strategy uses a colorimetric sensor based on gold nanoparticles and glutathione conjugate, with a limit of detection (LOD) of 1.2 nM. The sensor was successfully applied to different types of water samples, including natural mineral water, pure water, tap water, and river water. This study also reports on the detection of gold nanoparticles with an LOD of 0.14 μM, demonstrating the versatility of the sensor.

FS is a powerful analytical technique that has been widely used in food analysis due to its high sensitivity, selectivity, and nondestructive nature. It involves the excitation of a sample with a specific wavelength of light, followed by the emission of fluorescence light at longer wavelengths. RNT technology using FS has shown great potential for food analysis, which is a promising area that can help to ensure the safety and quality of food products. Li et al. [6] used a composite thin film made up of rhodamine B encapsulated in MOF-5 as a fluorescence sensor that responds to the volatile amines produced during the quality deterioration of pork. The study uses fluorescence spectra of the composite film combined with the partial least squares (PLS) algorithm to build quantitative and qualitative models for predicting the freshness indicator and classifying pork samples, respectively. The results indicate high accuracy in predicting and classifying the freshness of pork samples. Additionally, Li et al. [7] presented a new method for detecting biothiols based on the competitive modulation of gold nanoclusters and Hg^2+^ ions, encapsulated in a zeolite imidazole framework for predesigned aggregation-induced luminescence emission. The developed fluorescence strategy has a sensitive and specific response to trace amounts of biothiols, providing a promising method for quantifying biothiols in serum, which could promote progress in disease diagnosis.

NIR measures the absorption of light in the near-infrared range (from about 780 to 2500 nm) by a sample. It is a mature nondestructive analytical technique that combines chemometrics and has become a widely used analytical tool in the food industry. Bian et al. [8] proposed a weighted multiscale support vector regression method based on variational mode decomposition (VMD-WMSVR) for the UV–Vis spectral determination of rapeseed oil adulterants and the NIR spectral quantification of Rhizoma Alpiniae Offcinarum adulterants. The VMD-WMSVR method decomposes each spectrum into discrete mode components by VMD, then builds sub-models between each component and target value using SVR, and integrates the predictions of the sub-models by weighted average to obtain the final prediction. The proposed method shows potential in model accuracy compared with PLS and SVR. Hao et al. [9] combined visible–near infrared (Vis–NIR) spectroscopy with a 1D-CNN deep learning model for online detection of browning in Yali pears. The method achieved 100% prediction accuracy for healthy and browned pears in the test set. The results indicate that the combination of Vis–NIR spectroscopy and the 1D-CNN discriminant model can be used for online detection of browning in Yali pears.

Hyperspectral imaging technology provides both spatial and spectral information about a sample, which allows for more comprehensive analysis and visualization of the sample’s properties. This technology is more popular than NIR spectroscopy in many fields. Dong et al. [10] proposed a method to predict the content of alanine in beef quickly and nondestructively using near-infrared hyperspectral imaging (NIR-HSI) combined with two-dimensional correlation spectroscopy (2D-COS) analysis. The study identifies local sensitive variables related to Ala content by analyzing the sequence of chemical bond changes caused by synchronous and asynchronous correlation spectrum changes in 2D-COS. Simplified linear, nonlinear, and artificial neural network models were developed, and the PLSR model based on effective wavelengths was found to be the most effective. The results demonstrate that 2D-COS combined with NIR-HSI can be used as an effective method to monitor Ala content in beef.

Out of the ten papers published in this Special Issue, it is evident that all of them utilized chemometrics to process the analytical signal generated by various sensors, analyze large datasets, and extract valuable information. Moreover, machine learning algorithms were used to achieve precise qualitative and quantitative analysis. Notably, some researchers employed AI algorithms such as CNN to develop discriminant models. Therefore, in addition to developing high-performance sensor equipment, integrating the latest AI algorithms for deep mining of large sensor data can significantly benefit RNT technology-based biosensors for food analysis.

## References

[1] Ren G., Zhang X., Wu R., Yin L., Hu W., Zhang Z. (2023). Rapid Characterization of Black Tea Taste Quality Using Miniature NIR Spectroscopy and Electronic Tongue Sensors. Biosensors.

[2] Zhao C., Ma J., Jia W., Wang H., Tian H., Wang J., Zhou W. (2022). An Apple Fungal Infection Detection Model Based on BPNN Optimized by Sparrow Search Algorithm. Biosensors.

[3] Peng S., Wang A., Lian Y., Jia J., Ji X., Yang H., Li J., Yang S., Liao J., Zhou S. (2022). Technology for Rapid Detection of Cyromazine Residues in Fruits and Vegetables: Molecularly Imprinted Electrochemical Sensors. Biosensors.

[4] Lian Y., Wang A., Peng S., Jia J., Zong L., Yang X., Li J., Zheng R., Yang S., Liao J. (2022). Optimization of Sensors Data Transmission Paths for Pest Monitoring Based on Intelligent Algorithms. Biosensors.

[5] Gu Y., Jiao L., Cao F., Liu X., Zhou Y., Yang C., Gao Z., Zhang M., Lin P., Han Y. (2022). A Real-Time Detection Method of Hg^2+^ in Drinking Water via Portable Biosensor: Using a Smartphone as a Low-Cost Micro-Spectrometer to Read the Colorimetric Signals. Biosensors.

[6] Li J., Zhang N., Yang X., Yang X., Wang Z., Liu H. (2022). RhB@MOF-5 Composite Film as a Fluorescence Sensor for Detection of Chilled Pork Freshness. Biosensors.

[7] Li S., Wan Y., Li Y., Liu J., Pi F., Liu L. (2023). A Competitive “On-Off-Enhanced On” AIE Fluorescence Switch for Detecting Biothiols Based on Hg^2+^ Ions and Gold Nanoclusters. Biosensors.

[8] Bian X., Wu D., Zhang K., Liu P., Shi H., Tan X., Wang Z. (2022). Variational Mode Decomposition Weighted Multiscale Support Vector Regression for Spectral Determination of Rapeseed Oil and Rhizoma Alpiniae Offcinarum Adulterants. Biosensors.

[9] Hao Y., Li X., Zhang C., Lei Z. (2023). Online Inspection of Browning in Yali Pears Using Visible-Near Infrared Spectroscopy and Interpretable Spectrogram-Based CNN Modeling. Biosensors.

[10] Dong F., Bi Y., Hao J., Liu S., Lv Y., Cui J., Wang S., Han Y., Rodas-González A. (2022). A Combination of Near-Infrared Hyperspectral Imaging with Two-Dimensional Correlation Analysis for Monitoring the Content of Alanine in Beef. Biosensors.

